# Dephosphorylation of Caveolin-1 Controls C-X-C Motif Chemokine Ligand 10 Secretion in Mesenchymal Stem Cells to Regulate the Process of Wound Healing

**DOI:** 10.3389/fcell.2021.725630

**Published:** 2021-11-01

**Authors:** Panpan Wang, Yingji Zhao, Juan Wang, Zhiying Wu, Bingdong Sui, Xueli Mao, Songtao Shi, Xiaoxing Kou

**Affiliations:** ^1^South China Center of Craniofacial Stem Cell Research, Hospital of Stomatology, Sun Yat-sen University, Guangdong Provincial Key Laboratory of Stomatology, Guangzhou, China; ^2^Department of Microbiology, Zhongshan School of Medicine, Key Laboratory for Tropical Diseases Control of the Ministry of Education, Sun Yat-sen University, Guangzhou, China; ^3^Key Laboratory for Stem Cells and Tissue Engineering, Sun Yat-sen University, Ministry of Education, Guangzhou, China

**Keywords:** phospho-caveolin-1, CXCL10, mesenchymal stem cells, wound healing, secretion

## Abstract

Mesenchymal stem cells (MSCs) secrete cytokines in a paracrine or autocrine manner to regulate immune response and tissue regeneration. Our previous research revealed that MSCs use the complex of Fas/Fas-associated phosphatase-1 (Fap-1)/caveolin-1 (Cav-1) mediated exocytotic process to regulate cytokine and small extracellular vesicles (EVs) secretion, which contributes to accelerated wound healing. However, the detailed underlying mechanism of cytokine secretion controlled by Cav-1 remains to be explored. We show that Gingiva-derived MSCs (GMSCs) could secrete more C-X-C motif chemokine ligand 10 (CXCL10) but showed lower phospho-Cav-1 (p-Cav-1) expression than skin-derived MSCs (SMSCs). Moreover, dephosphorylation of Cav-1 by a Src kinase inhibitor PP2 significantly enhances CXCL10 secretion, while activating phosphorylation of Cav-1 by H_2_O_2_ restraints CXCL10 secretion in GMSCs. We also found that Fas and Fap-1 contribute to the dephosphorylation of Cav-1 to elevate CXCL10 secretion. Tumor necrosis factor-α serves as an activator to up-regulate Fas, Fap-1, and down-regulate p-Cav-1 expression to promote CXCL10 release. Furthermore, local applying p-Cav-1 inhibitor PP2 could accelerate wound healing, reduce the expression of α-smooth muscle actin and increase cleaved-caspase 3 expression. These results indicated that dephosphorylation of Cav-1 could inhibit fibrosis during wound healing. The present study establishes a previously unknown role of p-Cav-1 in controlling cytokine release of MSC and may present a potential therapeutic approach for promoting scarless wound healing.

## Introduction

Wound healing is a complex process involving increased proliferation, adhesion, and migration of cells from connective tissue and epithelium, inflammatory reactions, and extracellular matrix remodeling. Oral gingival and mucosal wounds heal faster with minimal scar formation than cutaneous wounds (Hu et al., 2014). Mesenchymal stem cells (MSCs), capable of self-renewal and differentiation into mesenchymal and non-mesenchymal lineages (Abe et al., 2012), are essential players in maintaining tissue homeostasis (Gronthos et al., 2006). As resident cells, MSCs from the gingival or skin play an important role during the wound healing process (Li et al., 2018). However, the detailed mechanism by which MSCs contribute to wound healing is still not fully understood.

Numerous secreted factors, including cytokines, growth factors, chemokines, contribute to the remolding process of cutaneous wound healing (Forbes and Rosenthal, 2014; Rani et al., 2015). MSCs secrete cytokines in a paracrine or autocrine manner to regulate immune response and tissue regeneration. Gingiva-derived MSCs (GMSCs) have a distinct neural crest origin and show characteristics of self-renewal, multipotent differentiation, and immunomodulatory capacities both *in vitro* and *in vivo* (Zhang et al., 2009; Xu et al., 2013). Our previous study showed that GMSCs displayed different secretion profiles compared with skin-derived MSCs (SMSCs) and produce a higher amount of interleukin-1 receptor antagonist (IL-1RA) to accelerate gingival and cutaneous wound healing in mice (Kou et al., 2018). However, the critical role of other cytokines secreted by MSCs in wound healing has not been elucidated except IL-1RA.

Previously, we found that MSCs use Fas/Fas-associated phosphatase-1 (Fap-1) complex combined with the caveolin-1 (Cav-1) to activate the exocytotic process, secrete cytokines, and small EVs (Kou et al., 2018). However, the detailed underlying mechanism of cytokine secretion controlled by Cav-1 remains obscure. Cav-1 is a scaffold protein and the main protein component of caveolae. It participates in various cellular functions, including transcytosis, potocytosis, signal transduction endocytosis, proliferation, and differentiation (Predescu et al., 1997; Zhang et al., 2021). Cav-1 tyrosine 14 (Y14) is the primary phosphorylation site targeted by Src (Huang and He, 2017). The increase of phospho-Cav-1 (p-Cav-1) versus total Cav-1 is related to the reducing plasma membrane-attached caveolae and a simultaneous increase of cytoplasmic vesicles (Zimnicka et al., 2016). Studies suggest that phosphorylation of Cav-1 is associated with the formation and internalization of caveolae. Previous research also revealed that Y14 phosphorylation regulates divergent actions on stem cells, such as migration, proliferation, and survival (Park and Han, 2009). We thus hypothesized that phosphorylation of Cav-1 might regulate the secretion process of MSCs. In the current study, we show that dephosphorylation of Cav-1 controls the cytokine secretion capacity of MSCs to secrete a higher amount of C-X-C motif chemokine ligand 10 (CXCL10) and may serve as a potential therapeutic approach for promoting scarless wound healing.

## Materials and Methods

### Animals

Female C57BL/6J, B6.MRL-Fas*^*lpr*^*/J (MRL/*lpr*, Fas-deficient), and B6.Cg-Cav1*^TM 1*Mls*^*/J (Cav-1 knockout) mice were purchased from the Jackson Laboratory (Bar Harbor, ME, United States) and Laboratory Animal Center of Sun Yat-sen University. Age-matched 8–10-week female mice from the same background were used in all experiments. All the animals were fed under specific pathogen-free conditions with an ambient temperature of 24°C, 55–65% relative humidity, and a 12:12 h light:dark cycle. Animal experiments were performed under protocols institutionally approved by Animal Care and Use Committee of Sun Yat-sen University (SYSU-IACUC-2021-000118).

### Reagents and Chemicals

Src kinase inhibitor 4-amino-5-(4-chlorophenyl)-7-(t-butyl) pyrazolo[3,4-d]pyrimidine (PP2) was purchased from Sigma-Aldrich (St. Louis, MO, United States). Recombinant mice tumor necrosis factor-α (TNF-α) (315-01A), interferon-γ (IFN-γ) (500-P119) was purchased from PeproTech (Rocky Hill, NJ, United States). *Fas* (sc-2931) and *Fap-1* (sc-145067) small interfering RNA (siRNA) was purchased from Santa Cruz Biotechnology (Santa Cruz, CA, United States). Lipofectamine^TM^ RNAiMAX transfection reagent (#13778030) was purchased from Thermo Fisher Scientific (Waltham, MA, United States). Mouse Cytokine Array Panel A (ARY006) was obtained from R&D system (Minneapolis, MN, United States). Mouse CXCL10 enzyme-linked immunosorbent assay (ELISA) Kit (ab214563) was purchased from Abcam (Cambridge, MA, United States). DeadEnd^TM^ Fluorometric TUNEL System (G3250) was purchased from Promega (Madison, WI, United States).

### Antibodies

Anti-Fas antibody (05-351) was purchased from Millipore (Burlington, MA, United States). Anti-Fap-1 (MBS242520) were purchased from MyBioSource (MyBioSource, CA, United States). Anti-CXCL10 (sc-374092), Cav-1 (7C8) antibodies were purchased from Santa Cruz Biotechnology (Santa Cruz, CA, United States). Anti-Caspase-3 (5A1E), Cleaved Caspase-3 (Asp175), Anti-p-Cav-1 (3251), proliferating cell nuclear antigen (PCNA) (2586s) were purchased from Cell Signaling Technology (Danvers, MA, United States). Anti-α-smooth muscle actin (α-SMA) antibody was purchased from eBioscience (14-9760-82). Anti-actin (A2066) antibody was purchased from Sigma-Aldrich (St. Louis, MO, United States). Collagen I (bs-10423R) and Collagen III (bs-0549R) were purchased from Bioss (Cambridge, MA, United States). Alexa Fluor 488, Alexa Fluor 568, and Alexa Fluor 647 secondary antibodies were purchased from Invitrogen (Carlsbad, CA, United States).

### Isolation of Mouse and Human Gingival, and Skin Mesenchymal Stem Cells

Murine GMSCs and SMSCs were isolated and cultured as reported by our previous study (Zhang et al., 2009; Xu et al., 2013; Kou et al., 2018). To put it simply, gingival tissue around the molars and dorsal skin tissues of mice were separated and minced. Then tissues were incubated in 2 mg/ml collagenase type I (Worthington Biochemical) and 4 mg/ml dispase II (Roche Diagnostics) in phosphate-buffered saline (PBS) for 1 h at 37°C. Single cell suspension was obtained by filtering through a 70 μm cell strainer (BD Biosciences). And then, cells were seeded in 10 cm diameter culture dish (Corning, NY, United States) in complete media containing alpha minimum essential medium (α-MEM, Invitrogen) supplemented with 20% fetal bovine serum (FBS), 2 mM L-glutamine, 55 mM 2-mercaptoethanol, 100 U/ml penicillin, and 100 μg/ml streptomycin (Invitrogen) at 37°C in 5% CO_2_. After an initial incubation for 48 h, the cultures were washed with PBS to eliminate the non-adherent cells. Colony-forming attached cells were cultured for another 7 days and then passaged with TrypLE^TM^ Express Enzyme (Thermo Fisher Scientific) once for further experimental use.

Human gingiva and skin were obtained as remnant or discarded tissues following routine procedures at Guanghua School and Hospital of Stomatology, Sun Yat-sen University, under the protocol approved by the Medical Ethics Committee of Hospital of Stomatology, Sun Yat-sen University (KQEC-2021-23-01). Tissues were minced aseptically and digested as previously mentioned. Cell suspension was filtered through a 70 μm cell strainer; plated in 100-mm culture dishes with α-MEM containing 15% FBS, 2 mM L-glutamine, 100 U/ml penicillin, and 100 μg/ml streptomycin, 10 mM L-ascorbic acid phosphate and cultured at 37°C in a humidified culture incubator with 5% CO_2_. The plastic-adherent cells were passaged and maintained in the complete growth medium. Cells from second to sixth passages were used in the experiments.

### Transfection of Small Interfering RNA in GMSCs

GMSCs were passaged on a culture plate and were 50–70% confluent at the time of transfection. *Fas*, *Fap-1*, and *Cav-1* siRNA (Santa Cruz Biotechnology) were used to treat the GMSCs according to the manufacturer’s instructions. Non-targeting control siRNA (Santa Cruz Biotechnology) was used as negative controls. Transfected GMSCs were incubated at 37°C for 48 h before further assay. The efficiency of siRNA knockdown was confirmed by western blotting analysis. Transfected cells were then treated with different concentrations of IFN-γ or TNF-α for 24 h, cells were used for protein extraction for western blotting, and the culture supernatants were used for ELISA.

### Cytokine Array and Enzyme-Linked Immunosorbent Assay Analysis

Total 0.4 × 10^6^ cells were seeded in 6-well plate, after the cell were attached, cells were serum deprived for 12 h and then treated with different stimuli for 48 h. Cell supernatant were collected and centrifuged at 2000 *g* for 10 min. And then supernatant were analyzed using a Mouse Cytokine Array Panel A Array Kit (R&D Systems) according to the manufacturer’s instructions. The results were scanned and analyzed using ImageJ software. CXCL10 levels of the cell culture supernatant were measured by ELISA kit according to the manufacturer’s protocol.

### Western Blotting

Total protein from cells and tissues were extracted using M-PER mammalian protein extraction reagent (Thermo Fisher Scientific, United States) with protease and phosphatase inhibitors cocktail (Thermo Fisher Scientific, United States). According to the manufacturer’s instructions, proteins from cytoplasmic and membrane fractions were extracted using Mem-PER Plus Membrane Protein Extraction Kit (Thermo Fisher Scientific). Briefly, adherent cells were lysed with a permeabilization buffer and centrifuged at 16,000 *g* for 15 min to separate the cytosolic and membrane proteins. Then, the cytosolic proteins were extracted from the supernatant, and the pellet was lysed again in a solubilization buffer to collect the membrane-associated proteins. Proteins were quantified using the BCA protein assay kit (Thermo Scientific, United States) following the manufacturer’s instructions. Then, 20 μg of proteins was separated by electrophoresis with 12% SDS-PAGE gel, and transferred onto a polyvinylidene difluoride membrane (Millipore, United States). Membranes were blocked with 5% BSA and 0.1% Tween 20 for 1 h, followed by overnight incubation with the primary antibodies at 4°C. The membranes were then incubated under room temperature for 1 h in species-related horseradish peroxidase-conjugated secondary antibody (Santa Cruz Biotechnology) and detected using SuperSignal West Pico Chemiluminescent Substrate (Thermo Fisher Scientific) and Biomax film (Bio-Rad); the sensitivity of this substrate was chosen to detect low-picogram amounts of protein in polyvinylidene difluoride membrane. β-actin was used as the internal loading control.

### Wound Healing in Mice

A 1.5 cm × 1.5 cm full-thickness wounds were created in the dorsal skin of the mice. Seven days after surgery, wounds were topically submucosally injected with 100 μl placebo (PBS) or 100 μl PP2 (50 mg/kg body weight) in PBS every other day. Digital photographs of the cutaneous wounds were taken, including a ruler for scale. The percentage of wound closure (expressed as a percentage of the initial wound area) was quantified on photographs using ImageJ public domain software (NIH, Bethesda, MD, United States). Mice were sacrificed and tissues around the wound were harvested 14 days later.

### Histology

For histological analyses, the excised skin from wound sites was fixed in 4% formalin overnight, dehydrated with a graded-alcohol series, embedded in paraffin, and sectioned perpendicularly to the wound surface into 5-μm-thick sections. Hematoxylin and eosin (H&E) staining was used for histological observations. Masson’s trichrome staining (Trichrome Stain LG Solution, Sigma-Aldrich) was applied to determine the degree of collagen maturity according to the manufactures’ instructions. For immunofluorescent staining, wound tissue was fixed in 4% paraformaldehyde overnight, dehydrated in 30% sucrose solution, embedded in OCT, and sectioned perpendicularly to the wound surface into 5-μm-thick sections.

### Immunofluorescence Study

Cells growing on coverslips were fixed in 4% paraformaldehyde, permeabilized with 0.2% Triton-X, blocked with blocking buffer (5% BSA, 0.3% Triton-100 in 1× PBS) for 1 h at RT. Subsequently, coverslips were incubated with the primary antibodies overnight and then stained with secondary antibodies in the dark at room temperature for 1 h. Then, slides were mounted with Vectashield mounting medium containing 4’,6-diamidino-2-phenylindole (DAPI) (Vector Laboratories Burlingame, United States). Isotype-matched control antibodies (Invitrogen) were used as negative controls. Images were acquired using a Carl Zeiss Airscan LSM-900 confocal microscope or Zeiss Elyra 7 with Lattice SIM and analyzed using the Zen 2.3 SP1 software, Blue Edition. Three-dimensional reconstruction of 2D *Z*-stack data from SIM-microscope was performed using IMARIS software (Oxford).

### TUNEL Assay

Excised skin slices were obtained and incubated with permeabilization solution (PBS; 0.1% Triton X-100; 0.1% sodium citrate) for 2 min on ice and then the slices were incubated with a TUNEL reaction mix for 30 min at 37°C, two additional washing steps were performed, and the cells were then stained with DAPI for a further 7 min. The number of TUNEL-positive stained cells was divided by the number of DAPI-stained nuclei. The stained cells were analyzed using Zeiss LSM 900 confocal microscope with a 63× oil immersion objective.

### Statistical Analysis

All data are shown as mean ± SD. Statistical analysis was performed by GraphPad Prism 8 (GraphPad Software, San Diego, CA, United States). Comparisons between two groups were analyzed using independent unpaired two-tailed Student’s *t*-tests. Differences between more than two groups were assessed by one-way analysis of variance (ANOVA) with Tukey’s *post hoc* test. *P*-values < 0.05 were considered statistically significant.

## Results

### GMSCs Produce and Secrete Higher Amounts of C-X-C Motif Chemokine Ligand 10 Than SMSCs

**FIGURE 1:**
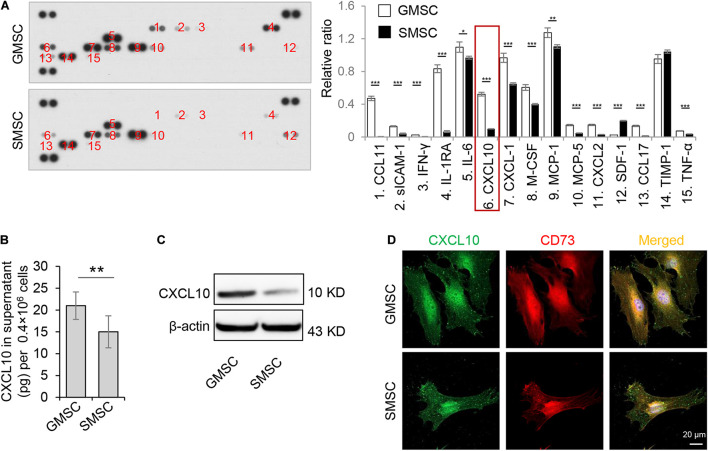
Murine GMSCs produce and secrete higher amounts of CXCL10 than SMSCs. **(A)** Cytokine array analysis of cytokines in cell culture supernatant from mouse GMSCs and SMSCs. Equal volume of cell culture supernatant were taken from 48 h incubation of 0.4 × 10^6^ cells. **(B)** ELISA analysis of CXCL10 in the culture supernatant of GMSCs and SMSCs. **(C)** Western blotting analysis showed that the protein expression of CXCL10 was higher in GMSCs than SMSCs. β-Actin was used as a protein loading control. **(D)** CXCL10 (green) co-expressed with the MSC marker CD73 (red) in GMSCs and SMSCs as analyzed by immunocytofluorescence staining. Scale bar, 20 μm. All results are representative of data obtained from at least three independent experiments. *P < 0.05, **P < 0.01, ***P < 0.001. Error bars are means ± SD. Data were analyzed using independent unpaired two-tailed Student’s *t*-tests.

In order to confirm the different secretion profiles between GMSCs and SMSCs, we compared the cytokine secretion pattern in the culture supernatant of murine GMSCs and SMSCs using the Proteome Profiler Cytokine Array. We found that in addition to previously reported IL-1RA (Kou et al., 2018), GMSCs also secreted a higher amount of CXCL10, a potent chemokine for activated T lymphocytes, compared to SMSCs (Figure 1A). ELISA analysis further confirmed that GMSCs secreted a higher level of CXCL10 (Figure 1B). In addition, western blotting analysis showed that both human and mouse GMSCs expressed elevated CXCL10 than SMSCs (Figure 1C and Supplementary Figure 1A). Next, we showed that CXCL10 was co-expressed with MSC markers CD73 in both GMSCs and SMSCs (Figure 1D).

### GMSCs Displayed Lower Caveolin-1 Phosphorylation Than SMSCs

**FIGURE 2:**
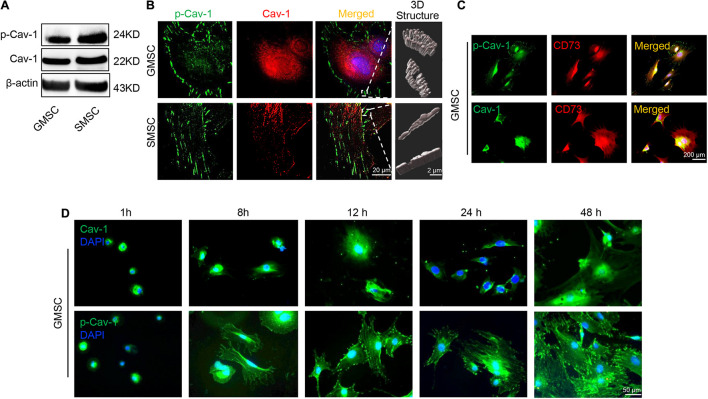
Murine GMSCs displayed lower Cav-1 phosphorylation than SMSCs. **(A)** Western blotting analysis showed the expression of p-Cav-1 and Cav-1 in GMSCs and SMSCs. **(B)** Higher resolution images of Cav-1 (red) and p-Cav-1 (green) in MSCs captured by SIM microscopy. Scale bar, 20 μm. Right panel, 3D reconstruction of 2D *Z*-stack data showed the different steric structures of p-Cav-1 in GMSCs and SMSCs. Scale bar, 2 μm. **(C)** Immunofluorescence analysis showed that p-Cav-1 or Cav-1 (green) co-localized with the MSC marker CD73 (red) in GMSCs. Scale bar, 200 μm. **(D)** Representative immunofluorescence staining images of p-Cav-1 and Cav-1 (green) in GMSCs at indicated time point after seeding to the culture plate. Time dependent p-Cav-1 translocation could be observed. Scale bar, 50 μm.

Our previous research revealed that GMSCs use the Fas/Fap-1/Cav-1 cascade to regulate cytokine and EV secretion (Kou et al., 2018). Previous research revealed that phosphorylation of Cav-1 is associated with the formation and internalization of caveolae and is involved in lots of cellular functions (Han et al., 2021). Tyrosine residue Y14 located at the NH2-terminus of Cav-1 protein, is the premier phosphorylation site (Shi et al., 2021). We thus speculated that change of Cav-1 Y14 phosphorylation status might contribute to Fas/Fap-1/Cav-1 cascade-controlled cytokine secretion of MSCs. Western blotting analysis revealed that Cav-1 Y14 phosphorylation in human and mouse GMSCs was lower than in SMSCs, while this trend was not observed in Cav-1 expression (Figure 2A and Supplementary Figure 1A). Semi-quantification analysis confirmed the significant difference of p-Cav-1 between GMSCs and SMSCs (Supplementary Figure 2). To further confirm the difference of Cav-1 phosphorylation between GMSCs and SMSCs, we performed immunofluorescent staining and used super-resolution structured illumination microscopy (SIM) to show that despite similar membrane located p-Cav-1 signal in both GMSCs and SMSCs, cytoplasmic p-Cav-1 was weaker in human and mouse GMSCs than SMSCs (Figure 2B and Supplementary Figures 1B, 3). On the other hand, although Cav-1 signal intensity did not show a significant difference between GMSCs and SMSCs, Cav-1 signal was nearly completely absent from p-Cav-1 in GMSCs, and lots of Cav-1 signals were colocalized with p-Cav-1 (indicated by merged yellow signal) in SMSCs (Figure 2B). In many cell types, caveolae are usually single or in chains or grape-like clusters (Stan, 2005). The three-dimensional reconstruction of SIM (3D-SIM) images further showed that the patterns of p-Cav-1 on the cell membrane of the two kinds of MSCs were different. Specifically, p-Cav-1 caveolae clusters on the GMSCs cell membrane were larger and plump, while the p-Cav-1 structure on SMSCs cell membrane was smaller and slender. 3D-SIM images further revealed that even the membrane p-Cav-1 has a different pattern.

Next, we confirmed that p-Cav-1 and Cav-1 signal was co-expressed with MSC marker CD73 (Figure 2C). Phosphorylation of Cav-1 is associated with the formation of caveolae, and we found most of the p-Cav-1 signal located on the cell edge. We, therefore, investigated the dynamic change of Cav-1 phosphorylation during the cell attachment process of suspended GMSCs. In the suspended GMSCs (1 h group), p-Cav-1 was dispersed throughout the intracellular compartment, where it was almost wholly co-localized with Cav-1. When the cells were attached, p-Cav-1 showed time-dependent translocation from the intracellular compartment to the plasma membrane, leading to increased p-Cav-1-labeled cell region of the GMSCs (Figure 2D).

### Dephosphorylation of Caveolin-1 Controlled C-X-C Motif Chemokine Ligand 10 Secretion in GMSCs

**FIGURE 3:**
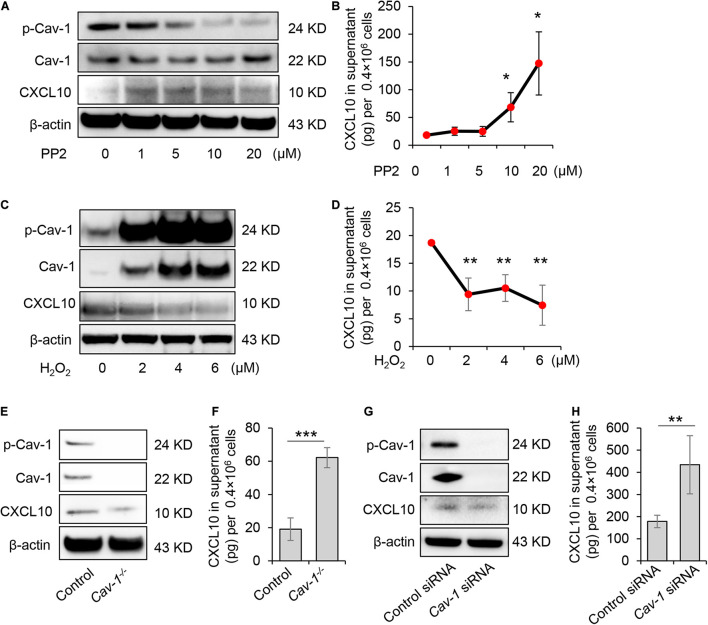
Dephosphorylation of Cav-1 controlled CXCL10 secretion in murine GMSCs. **(A)** Western blotting analysis showed cytoplasmic protein expression of p-Cav-1, Cav-1, and CXCL10 of GMSCs treated with different dose of PP2 for 2 h. **(B)** ELISA analysis of CXCL10 secretion in the culture supernatant from GMSCs treated with different dose of PP2 for 24 h. *P < 0.05 compared with control group. **(C)** Western blotting analysis showed cytoplasmic protein expression of p-Cav-1, Cav-1, and CXCL10 of GMSCs treated with different dose of H_2_O_2_ for 0.5 h. **(D)** ELISA analysis of CXCL10 secretion in the culture supernatant from GMSCs treated with different dose of H_2_O_2_ for 24 h. **P < 0.01 compared with control group. **(E)** Western blotting analysis showed absent expression of Cav-1 and p-Cav-1 and decreased cytoplasmic CXCL10 in *Cav-1^–^*^/^*^–^* GMSCs. **(F)** Knocking out of *Cav-1* elevated CXCL10 secretion into the culture supernatant of GMSCs as analyzed by ELISA. ***P < 0.001 compared with WT GMSCs control. **(G)** Western blotting analysis confirmed the efficiency of *Cav-1* siRNA, and showed that knocking down of *Cav-1* decreased cytoplasmic CXCL10 in GMSCs. **(H)** Knocking down of *Cav-1* elevated CXCL10 secretion in the culture supernatant of GMSCs as analyzed by ELISA. **P < 0.01 compared with GMSCs treated with control siRNA. All results are representative of data generated in at least three independent experiments. Error bars are means ± SD. Data were analyzed using independent unpaired two-tailed Student’s *t*-tests or one-way ANOVA.

GMSCs secreted a higher amount of CXCL10 and expressed decreased p-Cav in contrast to SMSCs (Figures 1, 2). Our previous study revealed that the complex of Fas/Fap-1/Cav-1 controlled EV/cytokine release (Kou et al., 2018). Here, we hypothesized that dephosphorylation of Cav-1 might control the secretion of CXCL10 in MSCs. Src kinase inhibitor 4-amino-5-(4-chlorophenyl)-7-(t-butyl) pyrazolo[3,4-d] pyrimidine (PP2) is an efficient Cav-1 phosphorylation inhibitor (Zimnicka et al., 2016). After PP2 treatment, GMSCs showed dose-dependent Cav-1 dephosphorylation on Y14, along with an increased accumulation of intercellular CXCL10 (Figure 3A). Meanwhile, CXCL10 secretion into the culture supernatant was elevated in a PP2 dose-dependent manner (Figure 3B). Acute H_2_O_2_ exposure can serve as a Cav-1 Y14 phosphorylation activator (Chen et al., 2005). After H_2_O_2_ treatment, GMSC showed a dose-dependent increase of Cav-1 phosphorylation on Y14, along with a decreased accumulation of intercellular CXCL10 (Figure 3C). Meanwhile, CXCL10 secretion into the culture supernatant was decreased in a H_2_O_2_ dose-dependent manner (Figure 3D). These data suggested that dephosphorylation of Cav-1 contributes to CXCL10 secretion in GMSCs.

To further confirm this phenomenon, GMSCs from *Cav-1*^–/–^ mice were used. Accompany with the absence of p-Cav-1, *Cav-1*^–/–^ GMSCs showed decreased intercellular accumulation of CXCL10, but secreted a higher amount of CXCL10 in the culture supernatant compared to WT control GMSCs (Figures 3E,F). Next, we used siRNA to knock down *Cav-1* and repress the phosphorylation of Cav-1 in GMSCs. We found that cytoplasmic CXCL10 expression was decreased, but CXCL10 secretion into the culture supernatant was elevated (Figures 3G,H). These data suggest that dephosphorylation of Cav-1 may control CXCL10 secretion in GMSCs.

### Fas Controlled Dephosphorylation of Caveolin-1 and C-X-C Motif Chemokine Ligand 10 Secretion

**FIGURE 4:**
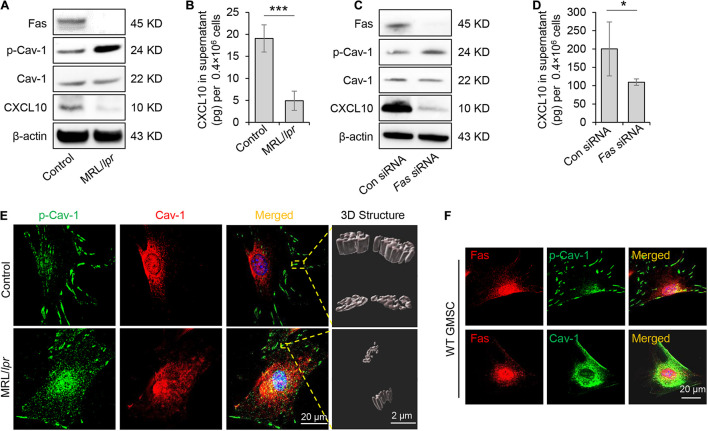
Fas controlled dephosphorylation of Cav-1 and CXCL10 release in murine GMSCs. **(A)** Western blotting analysis of p-Cav-1, Cav-1, and CXCL10 in GMSCs from *Fas*-deficient MRL/*lpr* mice. **(B)** Knocking out of *Fas* down-regulated CXCL10 secretion in GMSCs culture supernatant as analyzed by ELISA. ***P < 0.001 compared with WT GMSCs control. **(C)** Western blotting analysis confirmed the efficiency of *Fas* siRNA, and showed that knocking down of *Fas* up-regulated p-Cav-1 expression and reduced cytoplasmic CXCL10 in GMSCs. **(D)** Knocking down of *Fas* decreased CXCL10 secretion in the culture supernatant of GMSCs as analyzed by ELISA. *P < 0.05 compared with GMSCs treated with control siRNA. **(E)** Higher resolution images of Cav-1 (red) and p-Cav-1 (green) in MSCs captured by SIM microscopy. Scale bar, 20 μm. Right panel, 3D reconstruction of 2D *Z*-stack data showed the different steric structures of p-Cav-1 in GMSCs and MRL/*Lpr* GMSCs. Scale bar, 2 μm. **(F)** Immunocytofluorescence staining images of p-Cav-1, Cav-1, (green) and Fas (red) in GMSCs. Scale bar, 20 μm. All results are representative of data generated in at least three independent experiments. Error bars are means ± SD. Data were analyzed using independent unpaired two-tailed Student’s *t*-tests.

Previously study showed that Fas and Fap-1 complex bind to Cav-1 to control sEV/cytokine release (Kou et al., 2018). We thus speculated whether Fas and Fap-1 control dephosphorylation of Cav-1 and CXCL10 secretion. Western blotting showed that GMSCs from *Fas*-deficient MRL/*lpr* mice showed an elevated level of p-Cav-1 and decreased expression of CXCL10, along with the diminished secretion of CXCL10 to the culture supernatant (Figures 4A,B). When *Fas* expression was knocked down in GMSCs by siRNA, p-Cav-1 level in GMSCs was elevated along with repressive capacity of CXCL10 secretion into the culture supernatant (Figures 4C,D). These data suggested Fas controlled dephosphorylation of Cav-1 and secretion of CXCL10. Interestingly, we found that CXCL10 expression was declined in both *Fas*-deficient MRL/*lpr* GMSCs and *Fas* siRNA-treated GMSCs (Figures 4A,C), which suggested Fas may also control the expression of CXCL10.

To further confirm the Fas-controlled dephosphorylation of Cav-1, we performed immunofluorescent staining and used SIM microscopy to show that cytoplasmic p-Cav-1 signal was weaker in WT GMSCs than in *Fas*-deficient MRL/*lpr* GMSCs (Figure 4E). Although Cav-1 signal intensity did not show a significant difference between WT GMSCs and Fas-deficient MRL/*lpr* GMSCs, the Cav-1 signal showed marked clustering in the cytoplasm of Fas-deficient MRL/*lpr* GMSCs (Figure 4E). On the other hand, the Cav-1 signal was nearly completely absent from p-Cav-1 in WT GMSC, and most of Cav-1 signal was co-localized with p-Cav-1 (indicated by merged yellow signal) in *Fas*-deficient MRL/*lpr* GMSCs (Figure 4E). The 3D-SIM images further showed that p-Cav-1 structure on *Fas*-deficient MRL/*lpr* GMSCs membrane was smaller and slender than in the WT GMSCS (Figure 4E). The phenomenon was also observed in SMSCs (Figure 2B).

Next, we performed immunocytofluorescence staining to show the interaction of Fas with p-Cav-1 or Cav-1 in WT GMSCs. We found that the distribution patterns of Fas and p-Cav-1 were different in the cytoplasm and membrane of GMSCs (Figure 4F). Whereas Fas and Cav-1 signals showed similar distribution, and co-localization of Fas and Cav-1 was detected in the cytoplasm and membrane of GMSCs (Figure 4F). These data indicated Fas contribute to the dephosphorylation of Cav-1 and may bind to dephosphorylated Cav-1 to control CXCL10 secretion in MSCs (Figure 4F).

### Fas-Associated Phosphatase-1 Controlled Dephosphorylation of Caveolin-1 and C-X-C Motif Chemokine Ligand 10 Secretion

**FIGURE 5:**
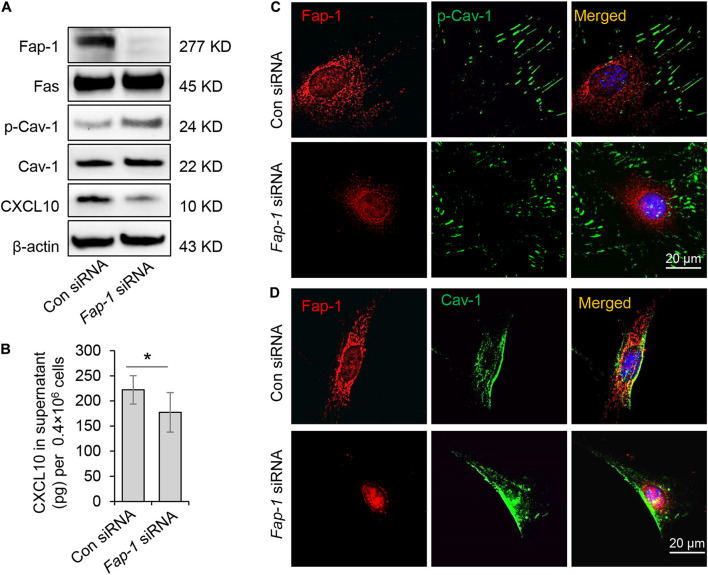
Fas-associated phosphatase-1 controlled dephosphorylation of Cav-1 and CXCL10 release in murine GMSCs. **(A)** Western blotting analysis was used to confirm the efficiency of *Fap-1* siRNA, and knocking down of *Fap-1* up-regulated p-Cav-1 expression but reduced cytoplasmic CXCL10. **(B)** ELISA analysis showed that knocking down of *Fap-1* decreased CXCL10 secretion in the culture supernatant of GMSCs. *P < 0.05. **(C)** Immunocytofluorescence staining images of p-Cav-1 (green) and Fap-1 (red) in WT control and *Fap-1* deficient GMSCs. Scale bar, 20 μm. **(D)** Immunocytofluorescence staining images of Cav-1 (green) and Fap-1 (red) in WT control and *Fap-1* deficient GMSCs. Scale bar, 20 μm. All results are representative of data generated in at least three independent experiments. Error bars are means ± SD. Data were analyzed using independent unpaired two-tailed Student’s *t*-tests.

Fas-associated phosphatase-1 is a protein tyrosine phosphatase and can binding to the cytosolic domain of Fas. It is also a critical component of the Fas/Fap-1/Cav-1 complex (Sato et al., 1995; Kou et al., 2018). To assess the functional role of Fap-1 in dephosphorylation of Cav-1 and CXCL10 release, we used siRNA to knock down *Fap-1* expression (Figure 5A). We found that *Fap-1* knockdown leads to increase of Cav-1 phosphorylation and reduced intercellular CXCL10 accumulation, along with a decrease of CXCL10 secretion into the culture supernatant (Figure 5B). In addition, *Fap-1* knockdown did not affect the expression of Fas and Cav-1 (Figure 5A). Immunofluorescence staining further confirmed that *Fap-1* knockdown lead to the increase of cytoplasmic p-Cav-1 (Figure 5C). In addition, Cav-1 signal showed marked clustering in the cytoplasm in Fap-1 knockdown GMSCs (Figure 5D).

### Tumor Necrosis Factor-α Up-Regulates Fas and Fas-Associated Phosphatase-1 to Dephosphorylate Caveolin-1 to Activate C-X-C Motif Chemokine Ligand 10 Secretion

**FIGURE 6:**
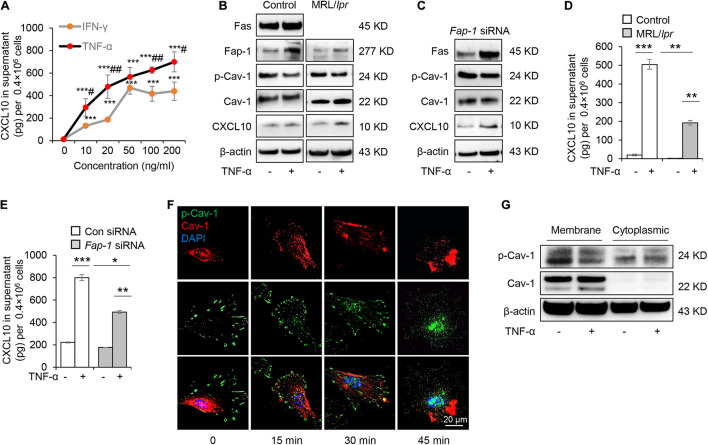
Tumor necrosis factor-αTNF-α up-regulates Fas and Fap-1 to dephosphorylate Cav-1 to activate CXCL10 secretion in murine GMSCs. **(A)** ELISA analysis of CXCL10 secretion in the culture supernatant from WT GMSCs treated with indicated dose of TNF-α or IFN-γ. ***P < 0.001, TNF-α or IFN-γ treated GMSCs versus control; ^#^*P* < 0.05, ^##^*P* < 0.01, TNF-α treated GMSCs versus IFN-γ treated GMSCs. **(B)** Western blotting analysis of Fap-1, Fas, p-Cav-1, Cav-1, CXCL10 protein expression of WT GMSCs and MRL/*Lpr* GMSCs with or without TNF-α (20 ng/ml) treatment. **(C)** Western blotting analysis of Fap-1, Fas, p-Cav-1, Cav-1, CXCL10 expression of control and *Fap-1* siRNA-treated GMSCs with or without TNF-α (20 ng/ml) treatment. **(D)** ELISA analysis of CXCL10 secretion in the culture supernatant from WT GMSCs, MRL/*lpr* GMSCs with or without TNF-α (20 ng/ml) treatment for 48 h. **P < 0.01, ***P < 0.001. **(E)** ELISA analysis of CXCL10 secretion in the culture supernatant from control and *Fap-1* siRNA-treated GMSCs with or without TNF-α (20 ng/ml) treatment for 48 h. *P < 0.05*, **P < 0.01, ***P < 0.001. **(F)** Immunocytofluorescence staining of GMSCs at various time points after TNF-α (20 ng/ml) treatment. p-Cav-1 (green) translocation from the cell membrane to the cytoplasm was indicated. Scale bar, 20 μm. **(G)** Western blotting analysis of protein expression of p-Cav-1, Cav-1 in cell membrane and cytoplasm. The results confirmed the translocation of p-Cav-1 from the cell membrane to cytoplasm upon TNF-α treatment. All results are representative of data generated in at least three independent experiments. Error bars are means ± SD. Data were analyzed using independent unpaired two-tailed Student’s *t*-tests.

Mesenchymal stem cells function can be elicited by inflammatory cytokines such as TNF-α and IFN-γ. We, therefore, speculated whether TNF-α and IFN-γ could promote the secretion of CXCL10 by MSCs. We found that both TNF-α and IFN-γ significantly enhanced the secretion of CXCL10 in the culture supernatant in a dose-dependent manner (Figure 6A). Furthermore, TNF-α was more efficient to induce the secretion of CXCL10 than IFN-γ, so we used TNF-α in the following experiments. To test whether TNF-α affects Fas/Fap-1 controlled Cav-1 dephosphorylation and Cav-1 dephosphorylation-related CXCL10 secretion, we showed that TNF-α treatment up-regulated the expression of Fas or Fap-1 but not Cav-1 in WT GMSC, MRL/*lpr* GMSCs, and *Fap-1* siRNA-treated GMSCs (Figures 6B,C), which was consistent with our previous study (Kou et al., 2018). In addition, TNF-α treatment induced a marked decrease of Cav-1 phosphorylation in WT GMSC but only slightly reduced p-Cav-1 level in MRL/*lpr* GMSCs and *Fap-1* siRNA-treated GMSCs (Figure 6B). Moreover, TNF-α treatment induced elevated intracellular accumulation of CXCL10 in MRL/*lpr* GMSCs and *Fap-1* siRNA-treated GMSCs, along with the attenuated secretion of CXCL10 into the culture supernatant when compared to WT control GMSCs (Figures 6D,E). These data suggested that TNF-α may serve as an activator to enhance CXCL10 release through dephosphorylation of Cav-1 and up-regulation of Fas and Fap-1.

Next, we used immunofluorescent staining to show that Cav-1 translocated to the cell membrane region at 15–45 min after TNF-α treatment. Simultaneously, p-Cav-1 signal was disappeared from the membrane and was dispersed throughout the cytoplasm after TNF-α treatment (Figure 6F). Western blotting further confirmed that TNF-α treatment decreased p-Cav-1 and increased Cav-1 expression in the cell membrane, but increased Cav-1 phosphorylation in the cytoplasm (Figure 6G). These data indicated that TNF-α treatment induces dephosphorylation of membrane p-Cav-1 to control Fas/Fap-1-mediated CXCL10 release.

### Dephosphorylation of Caveolin-1 Control Wound Healing Process

**FIGURE 7:**
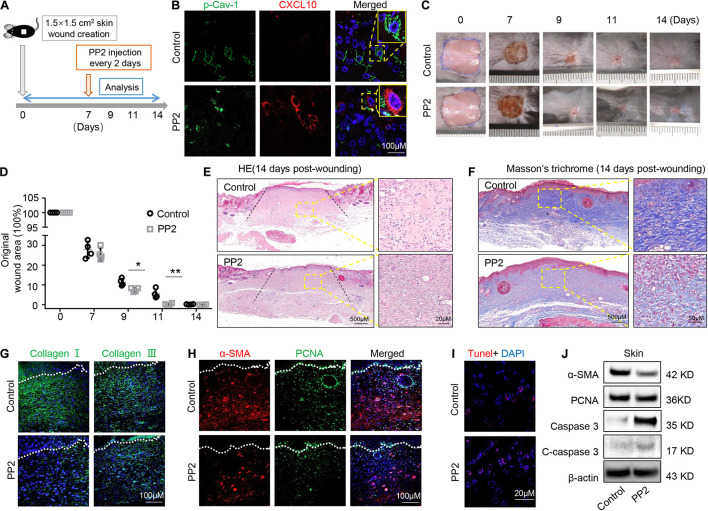
Dephosphorylation of caveolin-1 controls wound healing process in mice. **(A)** Scheme diagram illustrating the cutaneous wound procedure in C57BL/6 mice and treatment with PP2 or PBS. **(B)** Immunocytofluorescence staining of p-Cav-1 (green) and CXCL10 (red) in the wound area in control and PP2 treated group. Scale bar, 100 μm. Magnified images of the boxed region are showed in the upper right boxes. **(C)** Representative macroscopic images of cutaneous wound area in mice after treatment with and without PP2. **(D)** Quantification of wound area in mice over time. PP2 treatment could accelerate wound healing process on 9 and 11 days post-wounding compared to WT control mice. *P < 0.05, **P < 0.01. **(E)** Representative H&E image from cutaneous wounds in control and PP2 treated mice 14 days post-wounding. Scale bar, 500 μm. The black dash lines indicate the margin the unhealed wound area. The right box is a higher magnification of the boxed region in the image. Scale bar, 20 μm. **(F)** Evaluation of collagen maturity by Masson’s trichrome staining of wounds following treatment with PBS or PP2 at 14 days post-wounding. Scale bar, 500 μm. The right box is a higher magnification of the boxed region in the image. Scale bar, 50 μm. Note the bulky collagen fibers in control group. **(G)** Immunofluorescence analysis showed the expression of Collagen I and Collagen III (green) in the wound area in control and PP2 treated group. Scale bar, 100 μm. **(H)** Immunofluorescence analysis showed the staining of α-SMA (red) and PCNA (green) in the wound area in control and PP2 treated group. Scale bar, 100 μm. **(I)** TUNEL-positive apoptotic cells (red) in the wound area of PP2 treated group was significantly higher than in the control group. Scale bar, 20 μm. **(J)** Western blotting analysis of protein expression of α-SMA, PCNA, caspase 3, and cleaved-caspase 3 in wound area.

Oral gingival/mucosal wounds heal faster than cutaneous wounds and exhibit minimal scar formation (Hakkinen et al., 2000). We found that GMSCs showed lower expression of p-Cav-1 (Figure 2A) and higher production of CXCL10 than SMSCs (Figure 1C). CXCL10 is capable of inhibiting the migration of dermal fibroblasts by blocking their release from the substratum. We thus hypothesized whether dephosphorylation of Cav-1 and CXCL10 production could promote cutaneous wound healing. Src kinase inhibitor PP2 was locally injected around the wound sites every 2 days, 7 days after wounding. We found that PP2 injection decreased Cav-1 phosphorylation and promoted CXCL10 production around the cutaneous wound area (Figures 7A,B). More importantly, mice treated with PP2 showed accelerated wound closure at 9 and 11 days post-wounding compared to control mice (Figures 7C,D). H&E and Masson’s trichrome staining showed that almost whole epithelialization and excessive collagen formation were detected 14 days post-wounding, while PP2 injection reduced massive collagen deposition to a normal level (Figures 7E,F and Supplementary Figure 4). Immunofluorescent staining of Collagen I and Collagen III confirmed that PP2 treatment diminished collagen deposition at the wound areas (Figure 7G).

Since α-SMA is considered as a marker of scaring (Shinde et al., 2017). We used immunofluorescent staining to show that PP2 treatment leads to reduced expression of α-SMA and PCNA at the wound areas (Figure 7H), contributing to the down-regulation of hyperproliferative fibrosis during wound healing. Fibroblasts exhibit apoptotic phenotypes during wound heals but remained active in hypertrophic scars. Promoting the apoptosis of hyperproliferative scar fibroblasts contributes to the therapeutic effect of anti-scar drugs (Shao et al., 2020). Using TUNEL staining, we show that PP2 also increased cell apoptosis during wound healing (Figure 7I). Western blotting analysis confirmed that PP2 injection reduced the expression of α-SMA and PCNA, and increased the expression of apoptotic marker cleaved-caspase 3 in wound tissue (Figure 7J).

## Discussion

Adult cutaneous wound healing often ends up with cutaneous scarring, compromising the functional mobility and cosmetic outcomes (Zhang et al., 2016). However, like fetal wounds, oral gingival/mucosal wounds healing are characterized by markedly reduced inflammation, rapid re-epithelialization, and minimal scar formation (Hakkinen et al., 2000). Previous studies contribute this phenomenon to oral microbiota stimulating wound healing, the moist environment, and growth factors present in saliva etc. (Larjava et al., 2011). The present study unrevealed the function of Y14 phosphorylated Cav-1, which acts as a switch to regulate CXCL10 secretion in MSCs and mediate the fibrosis process of wound healing.

Mesenchymal stem cells isolated from different tissues exhibit different properties. They exert immunomodulatory effects by both cell-to-cell contacts and secreting biologically active substances, growth factors, cytokines, chemokines, and exosomes (Rani et al., 2015). The cytokine secretion profile of MSCs was found to be depend on the cell origin but not on the individuality of the donors (Park et al., 2009). The multitude of cytokines and growth factors secreted from MSCs are known to confer multifunctional functionality (Ivanova-Todorova et al., 2009). Gingiva is a unique oral tissue attached to the alveolar bone of tooth sockets, recognized as a biological mucosal barrier and a distinct component of oral mucosal immunity (Mao et al., 2017). Besides several unique stem cell-like properties, 90% GMSCs were derived from cranial neural crest cells and showed a superior capacity to differentiate into neural cells and chondrocytes and induce activated T-cell apoptosis (Zhang et al., 2009; Xu et al., 2013). Our previous study reported that GMSCs secrete higher amounts of sEVs and IL-1RA (Kou et al., 2018). The present study focus on Cav-1 dephosphorylation controlled MSCs secretion and found that besides IL-1RA, GMSCs also secreted a higher amount of CXCL10.

C-X-C motif chemokine ligand 10, also named IP-10, is a potent chemokine for activated T lymphocytes and regulates cell proliferation, apoptosis, and angiogenesis in infectious and inflammatory diseases and cancer (Liu et al., 2011). It is produced in the late stage of wound healing, becoming evident 4 days after wounding (Engelhardt et al., 1998). This pleiotropic molecule exerting potent biological functions during the whole process of wound healing: prolongs the granulation phase (Shiraha et al., 1999), and inhibits angiogenesis (Bodnar et al., 2009). These studies suggest that CXCL10 production represses cell proliferation and vessel formation. On the other hand, during the proliferation and remodeling phase of wound healing, excessive angiogenesis and over-proliferation, and migration of fibroblasts lead to scar formation (Rees et al., 2015). Here, we found that GMSCs secreted more CXCL10 into the cell culture supernatant, which may contribute to the scarless wound healing in gingiva. Furthermore, we showed that injection of Cav-1 phosphorylation inhibitor at the late stage of wounding promotes CXCL10 production at the wound area and accelerated cutaneous wound healing, repressed massive collagen formation and angiogenesis. Our finding was consistent with previous studies that CXCL10 inhibits the formation of fibrosis in healing infarct tissues (Saxena et al., 2014). To sum up, control the production of CXCL10 at the late stage of wound healing could be an attractive approach for limiting scar formation.

Along with the difference in CXCL10 secretion, we also found that more Cav-1 was dephosphorylated in GMSCs compared with SMSCs. Cav-1 drives the formation of flask-shaped membrane invaginations known as caveolae that participate in signaling, clathrin-independent endocytosis, and mechanotransduction (Tiwari et al., 2016). Cav-1 phosphorylation has been associated with cellular processes such as focal adhesion dynamics, cell migration and invasion, cancer cell metabolism, and response to mechanical, oxidative stress (Wong et al., 2020). Adhesion-dependent caveolar endocytosis is genuinely dependent on Cav-1 phosphorylation. However, controversial results could be found whether adhesion could regulate p-Cav-1 levels (del Pozo et al., 2005; Buwa et al., 2021). Here we found that p-Cav-1 showed time-dependent translocation from the intracellular compartment to the plasma membrane during cell attachment, which suggested that the adhesion process could regulate p-Cav-1 translocation. Several studies have confirmed the primary function of Cav-1 phosphorylation on Y14-mediated endocytosis, such as albumin, insulin (Sverdlov et al., 2007; Zimnicka et al., 2016). However, the relationship of Cav-1 phosphorylation on cell secretion is still unknown. Previous studies demonstrated that p-Cav-1 is involved in the process of insulin secretion by interacting with cell division cycle in pancreatic β-cells, which comprise an integral part of the insulin secretion vesicles (Haddad et al., 2020). Our previous study showed that MSCs secrete cytokines and EVs *via* the Fas/Fap-1/Cav-1 complex (Kou et al., 2018). The present study went further to reveal that dephosphorylation of Cav-1 acts as a switch in controlling cytokine CXCL10 secretion in MSCs.

Dynamic changes in wound healing are paralleled by changes in abundance cytokines and growth factors. MSCs can function as immunomodulatory and anti-inflammatory component resources, and the inflammatory microenvironment, *vice versa*, may stimulate paracrine factor production to promote MSC-mediated tissue homeostasis (Zhang et al., 2009). Several soluble factors have been attributed to the cross-talk with MSCs and immune cells, such as interleukin-10, prostaglandin E2, NO, and indoleamine 2,3-dioxygenase (Spaggiari et al., 2008). TNF-α and IFN-γ, two important pro-inflammatory cytokines secreted by activated T cells, serve as critical feedback signal molecules in the cross-talk between immune cells and MSCs (Bernardo and Fibbe, 2013). In this study, we found that both IFN-γ and TNF-α dose-dependently activate CXCL10 secretion in GMSCs. In addition, we found that TNF-α serves as an activator to enhance CXCL10 release through dephosphorylation of Cav-1 and up-regulation of Fas and Fap-1. TNF-α may take effect by translocating Cav-1 and p-Cav-1 from the cell membrane region to the nuclear region. These results extended our knowledge about the interaction between MSCs and the immune microenvironment.

Oral gingival/mucosal wounds heal faster than cutaneous ones, with minimal scar formation (Hakkinen et al., 2000). Here we show that dephosphorylation of Cav-1 mediated CXCL10 release could regulate fibrosis of wound healing and may contribute to the minimal scar formation in gingival wound healing. Normal wound healing starts with hemostasis and inflammation, granulation and proliferation, and finally ends with wound remodeling (Perry et al., 2010). The fibrosis wound also undergoes physical contraction throughout the entire wound-healing process, which is believed to be mediated by contractile fibroblasts (myofibroblasts) that appear in the wound (Guo and Dipietro, 2010). Myofibroblasts are activated fibroblasts marked by α-SMA expression and stress fiber formation. In this study, α-SMA expression was reduced by PP2 treatment. In addition, we also found that PCNA was down-regulated, and the expression of cleaved caspase-3 was up-regulated. The primary manifestation of hypertrophic scar is fibroblasts remain hyperactive and proliferate continuously, resulting in excessive collagen synthesis and deposition (Shao et al., 2020). Here, PP2 injection repressed Cav-1 phosphorylation and promoted apoptosis in wound tissue, suggesting that the compound reduces the population of hypertrophic scar fibroblasts not only by inhibiting cell proliferation but also by inducing cell apoptosis. PP2 induced apoptosis was also consistent with previous studies that p-Cav-1 and Cav-1 regulate the process of apoptosis (Han et al., 2015). CXCL10 was used to alleviate fibrosis disease, such as bleomycin-induced pulmonary bleomycin-induced pulmonary fibrosis (Tager et al., 2004), and p-Cav-1 was also reported to be key player in the process of fibrosis (Shihata et al., 2017). Our results were consistent with previous studies and raise the possibility of new approaches to alleviate scar formation during wound healing.

The results of the present study for the first time showed that dephosphorylation of Cav-1 controls secretion of CXCL10 in MSCs. TNF-α serves as an activator to up-regulate Fas, Fap-1, and down-regulate p-Cav-1 to promote CXCL10 secretion. In addition, we found that dephosphorylation of Cav-1 may regulate the skin wound healing process by relieving collagen deposition. Although detail study is still needed to explore the molecular mechanisms of p-Cav-1 mediated MSCs secretion, our evidence suggests that the blockade of Cav-1 phosphorylation at Y14 in MSCs accelerates CXCL10 secretion and would be beneficial in scarless wound healing.

## Data Availability Statement

The raw data supporting the conclusions of this article will be made available by the authors, without undue reservation.

## Ethics Statement

The studies involving human participants were reviewed and approved by the Guanghua School and Hospital of Stomatology, Sun Yat-sen University. The patients/participants provided their written informed consent to participate in this study. The animal study was reviewed and approved by the Sun Yat-sen University Animal Care and Use Committee.

## Author Contributions

PW contributed to designing study plan, performing experimental procedures, and drafting and final approval of the manuscript. YZ, JW, and ZW contributed to cell and animal experiments, immunofluorescence stain, data acquisition, and manuscript writing. BS and XM contributed to performing experiments, data acquisition, and analysis and interpretation. SS and XK contributed to the project conception, experimental design, writing manuscript, and supervision. All authors approved the final version of the manuscript.

## Conflict of Interest

The authors declare that the research was conducted in the absence of any commercial or financial relationships that could be construed as a potential conflict of interest.

## Publisher’s Note

All claims expressed in this article are solely those of the authors and do not necessarily represent those of their affiliated organizations, or those of the publisher, the editors and the reviewers. Any product that may be evaluated in this article, or claim that may be made by its manufacturer, is not guaranteed or endorsed by the publisher.
