# Supplementary material for: Dephosphorylation of Caveolin-1 Controls C-X-C Motif Chemokine Ligand 10 Secretion in Mesenchymal Stem Cells to Regulate the Process of Wound Healing
Source: Front Cell Dev Biol. 2021 Nov 1;9:725630. doi: 10.3389/fcell.2021.725630 (PMC8592036; doi:10.3389/fcell.2021.725630)
Supplement: Supplementary file 1 [file Data_Sheet_1.PDF]

Supplementary Figure and Legend

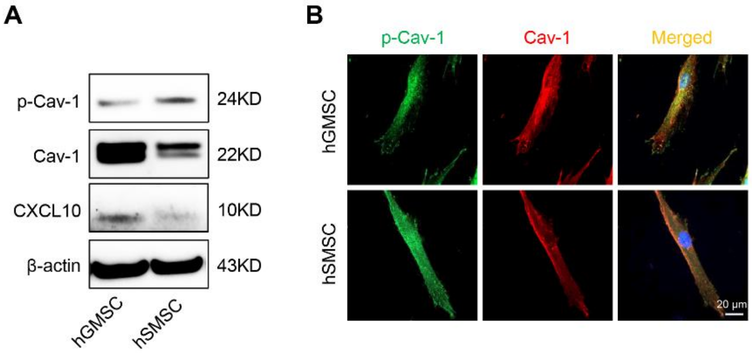

Supplementary Figure 1. Human GMSCs showed lower Cav-1 phosphorylation and expressed elevated CXCL10 than human SMSCs. (A) Western blotting analysis found that hGMSCs showed elevated expression of CXCL10 but lower Cav-1 phosphorylation than hSMSCs. (B) Immunocytofluorescence staining showed the staining of of p-Cav-1 (Green) or Cav-1 (Red) in human GMSCs and SMSCs. Scale bar, 20 μm.

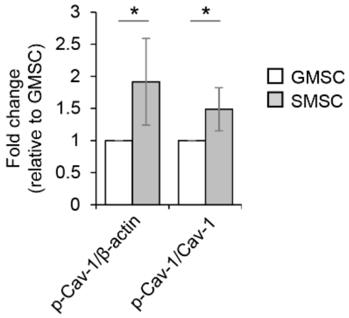

Supplementary Figure 2. Semi-quantification analysis of p-Cav-1 expression in GMSCs and SMSCs. *P < 0.05. Error bars are means ± SD.

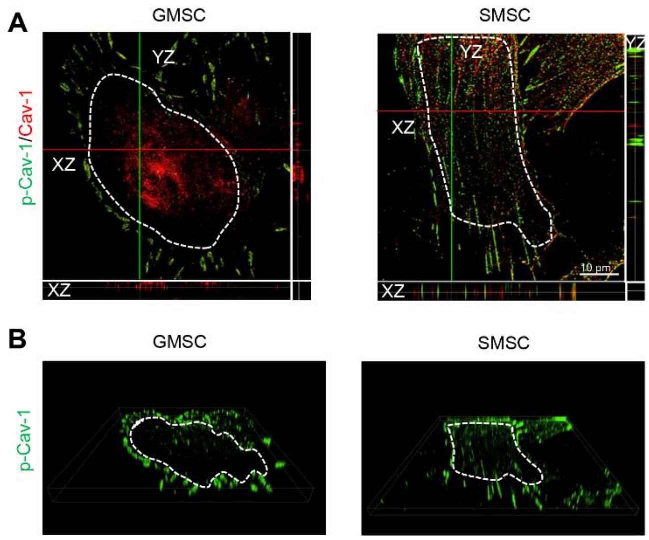

Supplementary Figure 3. Z-axis images to show the location of p-Cav-1 and Cav-1. (A) Z-axis sections of p-Cav-1 and Cav-1 immunofluorescent staining in GMSC and SMSC captured by super-resolution structured illumination microscopy (Zeiss Elyra 7 with Lattice SIM). XZ and YZ-axis images correspond to the position of the crossed line. Scale bar, 10 μm. (B) Iso-surface images p-Cav-1 and Cav-1 immunofluorescent staining in GMSC and SMSC generated with IMARIS software (Oxford). Scale bar, 10 μm. The white dash lines indicate the cytoplasm outline.

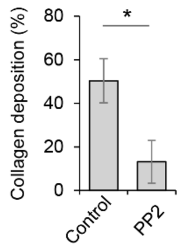

Supplementary Figure 4. Semi-quantification of collagen deposition on day 14 post-wounding. *P < 0.05. Error bars are means ± SD.

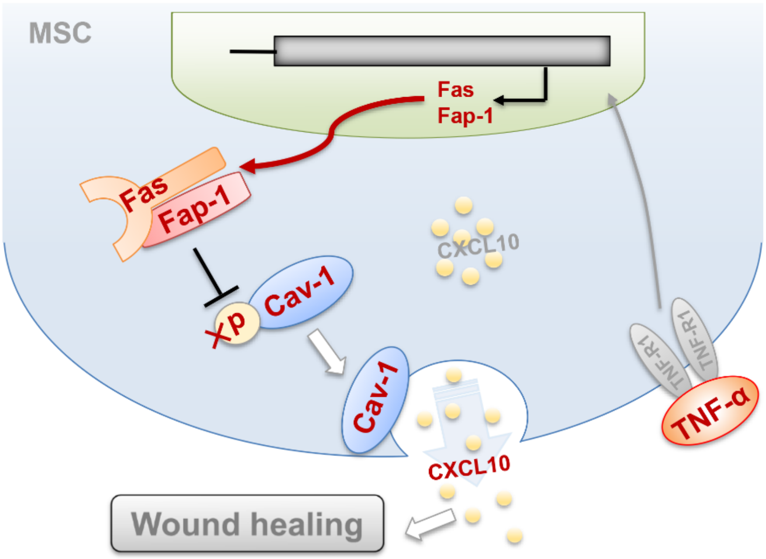

Supplementary Figure 5. Schematic drawing showed that TNF-α up-regulates Fas and Fap-1 expression to induce dephosphorylation of Cav-1, thereby controlling CXCL10 secretion in MSCs. CXCL10: C-X-C motif chemokine ligand 10; Fap-1: Fas associate phosphatase 1; Cav-1: Caveolin-1. p: phosphorylation.
